# Phonetic stability predicts individual differences in acoustic–phonetic cue weighting

**DOI:** 10.3758/s13423-026-02917-4

**Published:** 2026-10-09

**Authors:** Anne Marie Crinnion, James S. Magnuson, Emily B. Myers

**Affiliations:** 1 University of Connecticut, 2 Alethia Drive U-1085, Storrs, CT 06269 USA; 2 BCBL, Basque Center On Cognition, Brain and Language, Donostia/San Sebastián, Spain; 3 Ikerbasque, Basque Foundation for Science, Bilbao, Spain

**Keywords:** Speech perception, Cue weighting, Phonetic processing, Semantic priming

## Abstract

When processing speech, not every listener relies to the same degree on the various available types of information. It is unknown, however, whether differences in cue reliance reflect chronic or task-specific preferences of the individual. In a two-session study, we assessed how reliance on top-down (semantic) and bottom-up (acoustic–phonetic) cues differed across individuals in a cue-weighting task and how these patterns of cue weighting related to other independent measures of top-down and bottom-up cue use. We assessed semantic cue weighting by measuring semantic priming in a lexical decision task and we assessed the stability and gradience of listeners’ acoustic–phonetic sensitivity using a visual analogue scale task on two phonetic contrasts. We found that individuals who relied more on bottom-up cues when listening to sentences also had more consistent response patterns in our phonetic task. This finding suggests that individuals who rely more on acoustic–phonetic information when listening to sentences may do so because they have more stable low-level representations. Additionally, these results provide external validity as to the types of individual differences measures we assess, particularly in more naturalistic, sentence-listening contexts.

## Introduction

Listeners have many sources of information at their disposal to interpret the speech signal. Multiple acoustic–phonetic cues contribute to the identity of a sound (e.g., McMurray & Jongman, 2011; Morril, et al., 2015; Toscano & McMurray, 2012), cues that may be integrated probabilistically (e.g., Toscano & McMurray, 2012), or handled via a normalization process (e.g., Dupoux & Green, 1997). Adding to this picture, most psycholinguistic models allow for information above the level of the phoneme (e.g., lexical or semantic context) to influence interpretation of phoneme identity (e.g., Borsky et al., 1998; Crinnion et al., 2024b; Getz & Toscano, 2019; McClelland & Elman, 1986). Whatever the model one wishes to adopt of how multiple cues to phonemic identity are integrated, recent evidence suggests that there may be meaningful individual differences in *how* listeners weight cues during language processing.

When listeners are confronted by multiple cues to phoneme identity, group level data suggest that listeners integrate cues additively (e.g., Crinnion et al., 2024a; Kaufeld et al., 2020; see Martin, 2016, for discussion). However, other studies suggest great variability across individuals (Crinnion et al., 2024a). For instance, in Crinnion et al. (2024a), listeners heard sentences ending in a phonetically ambiguous word (e.g., “cup” vs. “cub” in the sentence *The lion sat near the cu[?]*). Three cues to that final word were manipulated; semantic information (lions relate more to cubs than cups) and two acoustic–phonetic cues: vowel length prior to the ambiguous token (longer vowels predict that a following stop is voiced), and rate of the preceding context (faster speech rates biases hearing the final stop as voiced; Heffner et al., 2013; Toscano & McMurray, 2015). While group-level data demonstrated additive patterns of sensitivity to all three cues, at the individual level, some listeners appeared to preferentially use acoustic–phonetic cues while others relied more on the semantic cue.

This result mirrors similar findings with other combinations of cues for sentence stimuli (e.g., morphosyntactic information and acoustic–phonetic information; Kaufeld et al., 2020), and also individual words (Giovannone & Theodore, 2021a, 2023; Lai et al., 2022). These observed individual differences seem to reflect in-the-moment processing differences across individuals, as they have been shown to correspond to differential eye movements as early as 200 ms after an ambiguous segment (Kaufeld et al., 2020). Additionally, individual cue weighting seems to be stable over time (Crinnion et al., 2024a; Giovannone & Theodore, 2023).

Listeners often show a trading relationship between the use of lower-level versus higher-level cues; those who strongly weight semantic information tend to weight acoustic–phonetic information less. Giovanonne and Theodore (2023) compared individual differences in reliance on lexical information across tasks. In a Ganong (1980) task, listeners heard tokens along a word-nonword continuum and identified the first phoneme (i.e., *[?]ift*, sampled from a gift-*kift continuum). They observed a strong trade-off between lexical and phonetic cues. They also observed this stable, negative relationship between signal (phonetic) and knowledge (lexical) cues in a *locally time-reversed speech task*: listeners who were more sensitive to reversals (i.e., correctly detecting a token with a reversal as different from one without) in words were less sensitive to them in nonwords, and vice versa. However, despite finding similar individual reliance on signal- versus knowledge-based cues across sessions, they found limited evidence for any such relationship across tasks.

Giovannone and Theodore (2021a, b) also found that individuals with weaker receptive language abilities tend to rely more on lexical versus phonetic information in a Ganong task. This suggests that individuals with weaker language abilities rely more on higher-level contextual information (at least when there is a high degree of conflict between cues), perhaps due to underlying weaknesses in acoustic–phonetic processing. Similarly, individuals with lower language abilities perform worse on speech in noise tasks (McLaughlin et al., 2018), and those who rely more on lexical information in a Ganong task perform worse on (at least some) speech-in-noise tasks (Lam et al., 2017). Thus, there is growing evidence that individuals reliably differ in the use of signal- versus knowledge-based cues, particularly in tasks where these compete (showing trading relationships).

But how do such differences arise, and how stable are they? Do individuals develop chronic balances of cue weighting that we would observe in any challenging speech task? In other words, do individual differences emerge due to specific task constraints (such that a listener might exhibit quite different weightings in different tasks), or do they vary unsystematically from task to task? In the current study, we tested whether individual differences in cue weighting (i.e., a reliance on acoustic–phonetic vs. semantic information) could be predicted by other tasks designed to assess sensitivity to acoustic–phonetic information (via the visual analogue scale task, or VAS) and to semantic processing (via semantic priming in a lexical decision task). We turn now to a discussion of these tasks.

A powerful way to measure acoustic–phonetic sensitivity is through the visual analogue scale (VAS) task, where listeners hear a token along a continuum, and give a continuous rating of the token (e.g., from most/p/-like to most/b/-like for a/p/-/b/continuum; Apfelbaum et al., 2022; Kapnoula et al., 2017; Kong & Edwards, 2016; Massaro & Cohen, 1983). VAS studies have revealed strong individual tendencies, with some individuals responding more categorically (giving ratings mainly near the endpoints, with a steep slope between categories) and others responding more gradiently (using the full scale in a more continuous fashion; Fuhrmeister & Myers, 2021; Kapnoula et al., 2017; Myers et al., 2024). Beyond observing individual response functions, we can assess to what degree participants respond similarly when presented with the same token, and use this “response consistency” as an index of the stability of their perceptions (Myers et al., 2024).

Response consistency has proved to be a more robust measure than gradience/slope in predicting proficiency on other speech and hearing tasks (e.g., Kim et al., 2025; Kutlu, et al., 2024; Myers et al., 2024). Higher response consistency in a native-language VAS task predicts better non-native speech sounds discrimination (Fuhrmeister et al., 2023; Honda et al., 2024). Degree of consistency has also been linked to brain structure (more consistent responders have less gyrification in temporal areas; Fuhrmeister & Myers, 2021). Additionally, listeners who are more consistent responders perform better in a speech-in-noise task (Myers et al., 2024). Thus, more consistent responders appear to have more precise access to acoustic–phonetic details in speech, with positive impacts on processing and learning.

Turning to semantic context, degree of semantic priming provides a key potential index of individual differences. There is mixed evidence as to the reliability of semantic priming effects (Kiang et al., 2013; Yap et al., 2016), but its moderate reliability makes it a suitable index. Thus, in the current study, we used semantic priming using lexical decision, where listeners decide whether a written item is a word or nonword when preceded by related versus unrelated primes. We utilized openly available items for which reliability has been previously established (Hutchison et al., 2013; Yap et al., 2016).

The goal of the current study was to characterize individual variation in reliance on semantic and acoustic–phonetic cues. We assessed this variability using a paradigm we refer to as a *cue-weighting task*, where listeners heard sentences and made decisions about the final word in the sentence (Crinnion et al., 2024a), while acoustic–phonetic and semantic cues to that final word were manipulated (see *Methods*). Two tasks served as measures of acoustic–phonetic sensitivity (*VAS)* and use of semantic information (*semantic priming*). We used stimulus materials that did not overlap across tasks to test the hypothesis that individual differences in sensitivity to acoustic–phonetic and semantic information would predict how listeners relied on these same sources of information when listening to sentences.

## Methods

### Participants

One hundred and five participants were recruited from Prolific (www.prolific.com) to reach a sample size of 100 participants after exclusions (47 identified as female, 55 as male, three unreported; age range: 18–35 years, mean age: 28 years) for the first day of data collection in which participants completed the cue-weighting task online (run through Gorilla; www.gorilla.sc; Anwyl-Irvine et al., 2020). This sample size was chosen because it was sufficiently large enough to detect individual differences in this task (see Crinnion et al., 2024a, for justification of sample size). We expected that 100 participants on day one would lead to 60–80 participants (after exclusions and attrition) on day two of testing, which is in line with sample sizes in similar studies (e.g., Giovanonne & Theodore, 2023).

Participants were paid $7.50 for day one and $10 for day two (consistent with the Connecticut state minimum wage at the time). Only participants who were 18–35 years of age, native speakers of North American English, with reported normal/corrected-to-normal vision, and normal hearing were included.

Five participants were excluded on day one after failing a headphone check meant to ensure use of headphones (Woods et al., 2017). The remaining 100 subjects were invited for a second session (1–7 days later), of whom 79 returned. Two participants failed the headphone check on day two and were excluded. Four people were excluded for using browsers other than Firefox or Chrome (Bridges et al., 2020, report best results analyzing reaction time data from those browsers). Three additional participants were excluded for having less than 80% accuracy in lexical decision. Three participants were excluded because their range of responses on the VAS task was less than 2 (see VAS task description below). Thus, the final dataset included data from 67 participants.

### Materials

#### Cue-weighting task

The cue-weighting task was taken from Crinnion et al. (2024a). In summary, in this task, participants listened to a total of 348 unique sentences. Participants responded by indicating the final word of the sentence by pressing one of two keys on the keyboard. Response options were always a voiced and voiceless word-final stop minimal pair (e.g., cub and cup).

Sentences varied along three dimensions: (1) semantic information, (2) vowel duration of the final word, and (3) the speaking rate of the sentence prior to the final word. Semantic information in a sentence was either voiced-biasing (e.g., The lion sat near the cu[p/b], where lion is more related to cub than cup) or voiceless-biasing (e.g., The plate sat near the cu[p/b], where plate is more related to cup than cub). There were three vowel durations, with the shortest promoting perception of a subsequent voiceless stop (e.g., cup) and the longest promoting perception of a subsequent voiced stop (e.g., cub). Finally, there were two sentence rates, one fast, which biases towards perception of a voiced-final word, and one slow, which biases towards perception of a voiceless-final word (Heffner et al., 2013). There were 29 sentence contexts (e.g., lions-plates is one sentence context). Thus, there were 29 sentence contexts, two semantic biases, three vowel lengths, and two rate biases, for a total of 348 unique sentences.

#### Visual analogue scale (VAS) task

Participants listened to items from two seven-step continua: one from *ba* to *da* and one from *sign* to *shine*. Stimuli were taken from Myers et al. (2024). The *ba-da* continuum was created from a Klatt synthesizer (Klatt, 1980), which changed the information contained in the first and second formants to create this continuum. The *sign-shine* continuum was created through waveform averaging in Praat (Boersma & Weenink, 2022). Natural recordings of both words (/sɑ͡ɪn/and/ʃɑ͡ɪn/) were recorded by a female native speaker of English. The initial fricatives were excised, normalized for length, and blended at proportions from 80 to 20%/s/, then re-appended to the/-ɑ͡ɪn/frame. Participants made a response after hearing each token by clicking on a number from 1 to 7 to indicate how much like one sound or the other the token was. For example, in the *shine*-*sign* continuum, participants clicked on a number from 1 to 7 after hearing a token, with 1 being the response for “the syllable that sounds ‘most shine-like’” and 7 being the response for “the syllable that sounds ‘most sign-like’.” Each participant heard 105 trials from one continuum (seven steps in each continuum, for 15 repetitions per step) in one block, and then the other continuum, for a total of 210 trials. The order of which continuum they heard first was counterbalanced across participants. Within each block, trial order was randomized across participants.

#### Semantic priming task

In this task, participants heard a prime word and then saw a written item, and responded with whether that item was either a word or a nonword. On a given trial, participants first saw a fixation cross for 1,000 ms, heard a prime word, and then saw a written item for 3,000 ms. They were instructed to press ‘F’ or ‘J’ on the keyboard (with button mapping counterbalanced across participants), depending on whether the written item was a word or a nonword. They then saw a progress bar that indicated how much of the experiment they had completed for 1,000 ms. After 12 practice trials, participants completed 300 trials, with breaks every 100 trials. 150 trials were nonword trials, and 150 trials were word trials. Of the 150 word trials, 75 pairs were related (e.g., AGRICULTURE-farm) and 75 pairs were unrelated (e.g., FEELING-farm). Each participant saw a target only one time, and presentation of each target with a related versus unrelated item was counterbalanced across participants. Stimulus pairs were selected from the Semantic Priming Project (Hutchison et al., 2013), and the auditory primes came from MALD (Tucker et al., 2019). Any pairs with homophones were excluded, and there was no overlap across pairs in surface forms or roots. Items were selected that exhibited the largest semantic priming effects in the Semantic Priming Project.

### Procedures

**Fig. 1:**
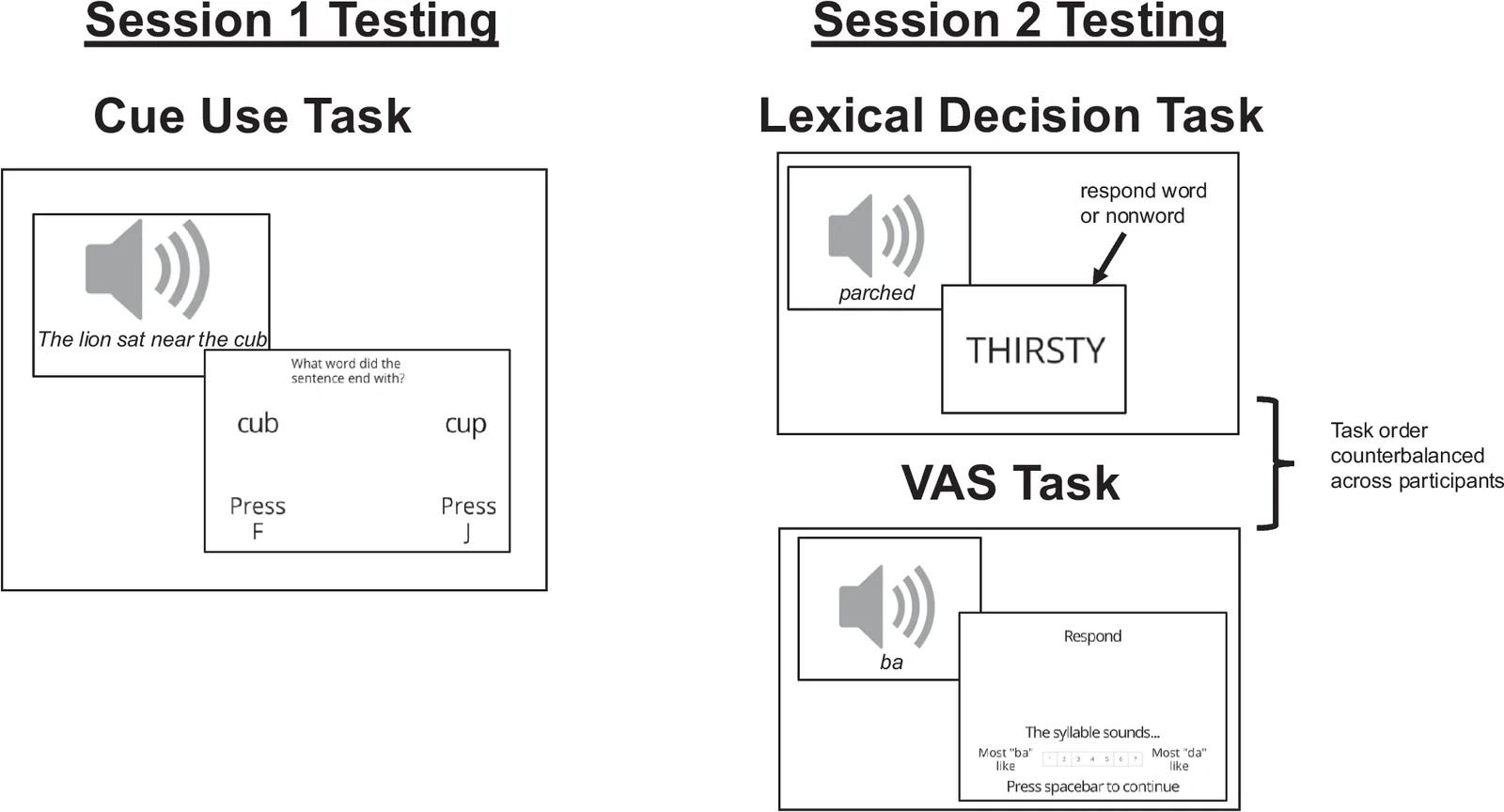
Schematic of the experimental tasks

Participants completed our three tasks over 2 days of testing (Fig. 1). The experiment was conducted online through Gorilla (www.gorilla.sc) and participants were recruited through Prolific (www.prolific.com). After providing informed consent, participants completed a headphone check to ensure headphone use (Woods et al., 2017). They then completed the cue-weighting task. The next day, participants who passed the headphone check were invited back to complete the VAS task and the semantic-priming task up to 1 week later. At the second session, they first provided informed consent and then completed the headphone check again. Participants were randomly assigned to complete either the VAS tasks (ba-da and shine-sign) first, or the semantic-priming task first. They then completed the other task. At the end of all tasks, participants provided demographic information.

### Analyses

We analyzed data for participants from whom we had data from all tasks (cue-weighting task, both VAS continua, and the semantic-priming task). All analyses were conducted in R (R Core Team, 2024). Unless otherwise specified, all mixed-effects models were run using the *afex* (Singmann et al., 2015) package, as this allowed us to obtain ANOVA-like model results (reporting chi-square values for the significance of model predictors).

#### Cue-weighting task

We fit a logistic mixed effects model predicting the proportion of voiced responses, using the same model structure as Crinnion et al. (2024a, b). Namely, the model structure was:

$$voicingcount \sim semantic\_bias * vowel\_length\_step * rate\_bias +$$

$$(semantic\_bias*vowel\_length\_step* rate\_bias | participant) +$$

$$(semantic\_bias * vowel\_length\_step * rate\_bias | item\_pair)$$

This model contained fixed effects for semantic bias, sentence rate, vowel length step, and their interactions. The model also contained random slopes and intercepts for all the effects and interactions above for both participants and items. We used the maximal model licensed by our design (Barr et al., 2013), and it was particularly important to retain random slopes by participant for semantic bias, vowel length step, and rate in order to estimate effects that represent the extent to which an individual relies on semantic and acoustic–phonetic (vowel length and rate) information, respectively (see Crinnion et al., 2024a, b).

#### VAS task

For each continuum, we fit a three-parameter logistic mixed effects model (four-parameter models did not converge) to model participants’ responses on a 7-point scale, similar to analysis approaches from Fuhrmeister and Myers (2021). The three parameters that we fit were the slope, the midpoint, and the upper asymptote of the logistic function both at the fixed (group) level and the random (subject) level. Models were fit using the *nlme* package (Pinheiro et al., 2023). We extracted the random slope for each subject, which served as the index of how gradiently or categorically the individual perceived the continuum. We also calculated response consistency as the negative of the average standard deviation of response across continuum steps, such that higher values indicated a response pattern of greater consistency (lower standard deviation).

#### Semantic priming task

For the semantic-priming task, we restricted the analysis of trials to only those where the target was a word and the participant correctly identified it as such. We excluded trials where participants’ reaction times were less than 150 ms or greater than 1,500 ms. We also excluded trials from two pairs of words that were incorrectly coded in the original Semantic Priming Project. As noted earlier, three participants with accuracy less than 80% were excluded. Mean accuracy was 97% for the included participants, with a range from 85 to 99%.

We ran a linear mixed-effects model predicting reaction time. This model contained fixed effects for the frequency of the target word (calculated from the English Lexicon Project; Balota et al., 2007), the length of the target word, the trial number, the spreadsheet, and the status of the prime (whether it was related or unrelated to the target word). The model also contained random intercepts and slopes for the status of the prime by subject and by target word. The individual slope for the effect of semantic relatedness served as the key measure of individual semantic context use.

#### Across-task relationships

Two analyses were performed to examine between-task relationships. To test whether semantic cue use in the priming task related to semantic cue use in the cue-weighting task, we calculated the Pearson correlation between the by-participant slope estimate for semantic bias in the cue use task and the by-participant slope estimate for the prime status in the semantic-priming task. To examine whether acoustic–phonetic sensitivity (both slope and consistency measures) in the VAS task related to acoustic–phonetic cue use in the cue-weighting task, we calculated the Pearson correlation between the by-participant slope estimate for vowel length step (instead of sentence rate), as vowel length was the biggest acoustic–phonetic effect (had the largest beta value among the acoustic–phonetic predictors in the models in Crinnion et al., 2024a) and each of our slope and consistency measures from our VAS task.

## Results

### Cue-weighting task

#### Group level

**Fig. 2:**
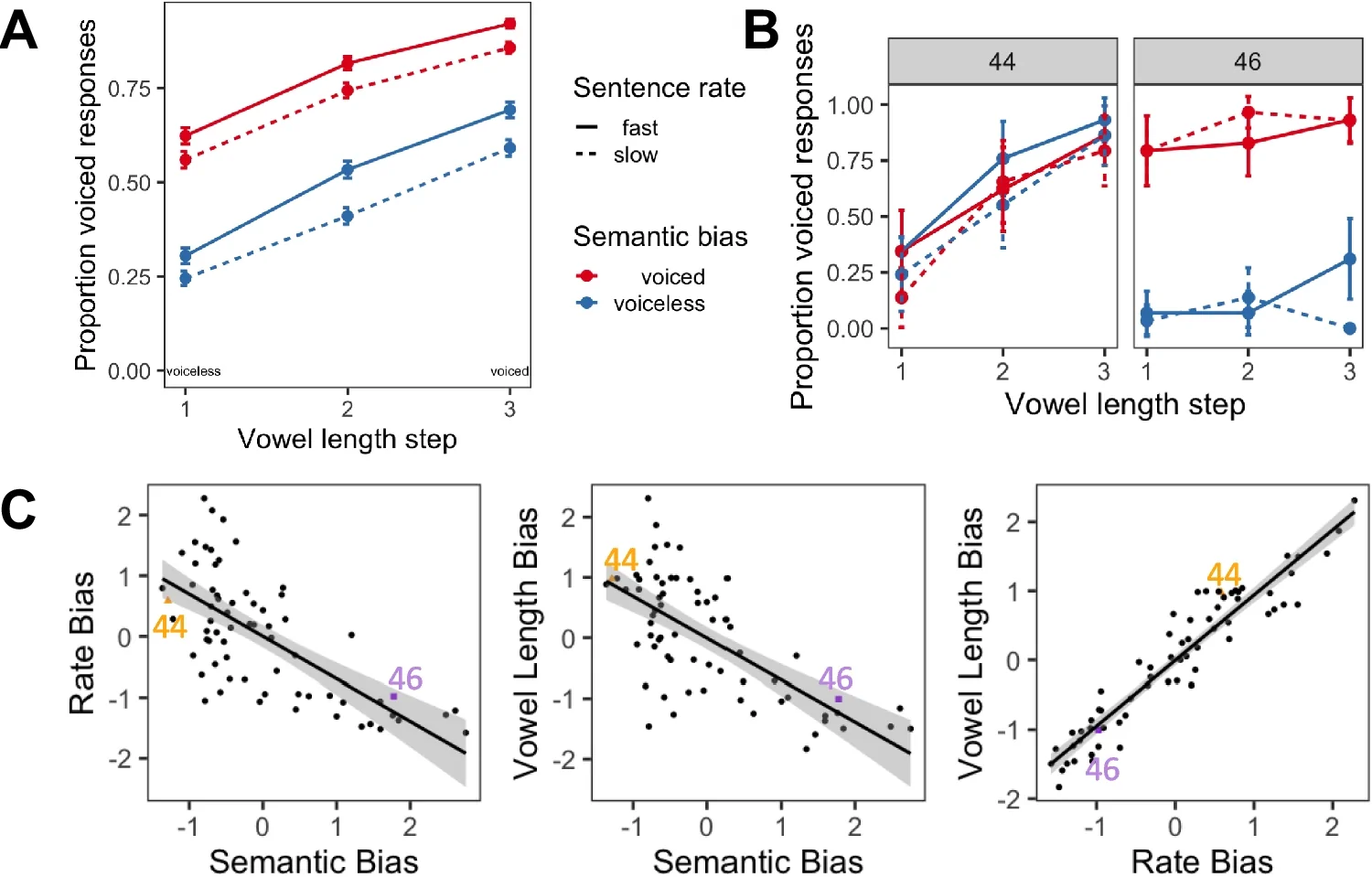
A. Results from the cue-weighting task in the aggregate. The x-axis shows the vowel length step, ranging from shortest (most voiceless-like) to longest (most voiced-like). The y-axis represents the proportion of voiced responses (e.g., the proportion of times participants responded that they heard *cub* as opposed to *cup*). The colors represent the semantic bias and the line type indicates the sentence rate bias, either fast (voiced biasing) or slow (voiceless biasing). **B.** Data from two representative participants. Participant 44 exhibits a strong influence of vowel length bias, and Participant 46 exhibits a strong influence of semantic bias. **C.** Relationships between cues. All values represent the scaled by-subject slopes from our mixed-effects models. We observe a strong negative relationship between semantic bias and both rate and vowel length bias, whereas we observe a strong positive relationship between rate and vowel length bias. Participant 44 is highlighted in orange, and Participant 46 is highlighted in purple

As shown in Fig. 2a, the group-level analysis directly replicated the findings from Crinnion et al. (2024a). We found significant effects of semantic bias (χ2 = 44.17, *p* <.001, with more voiced responses after voiced-biasing sentences), vowel length (χ2 = 85.76, *p* <.001, with more voiced responses after longer vowels), and sentence rate (χ2 = 49.33, *p* <.001, with more voiced responses after faster sentences). Additionally, we found a significant sentence rate by vowel length interaction (χ2 = 13.57, *p* <.001), such that the effect of rate was larger for longer vowels than for shorter vowels. Using the *emtrends* function from *emmeans* (Lenth, 2025), we found a significant difference in model slopes for rate conditions (fast vs. slow) across our vowel length steps (estimate:.223, *SE* =.052, z.ratio = 4.309, *p* <.001), with a steeper slope for faster rate.

#### Individual level

At the individual level there was considerable variability in cue weighting. Figure 2b shows data from two representative participants: Participant 44 (more reliant on vowel length) and Participant 46 (more reliant on semantic information). Figure 2c shows a trade-off between individual differences in reliance on semantic versus acoustic–phonetic cues (vowel length, sentence rate; replicating Crinnion et al., 2024a). As a reminder, cue reliances were estimated based on the by-participant slopes for each effect of interest. Semantic bias was negatively correlated with vowel length (r = -.69, *t*(65) = −7.608, *p* <.001) and rate (r = -.68, *t*(65) = −7.544, *p* <.001), whereas vowel length bias and rate were highly positively correlated (r =.95, *t*(65) = 24.303, *p* <.001).

### VAS task

**Fig. 3:**
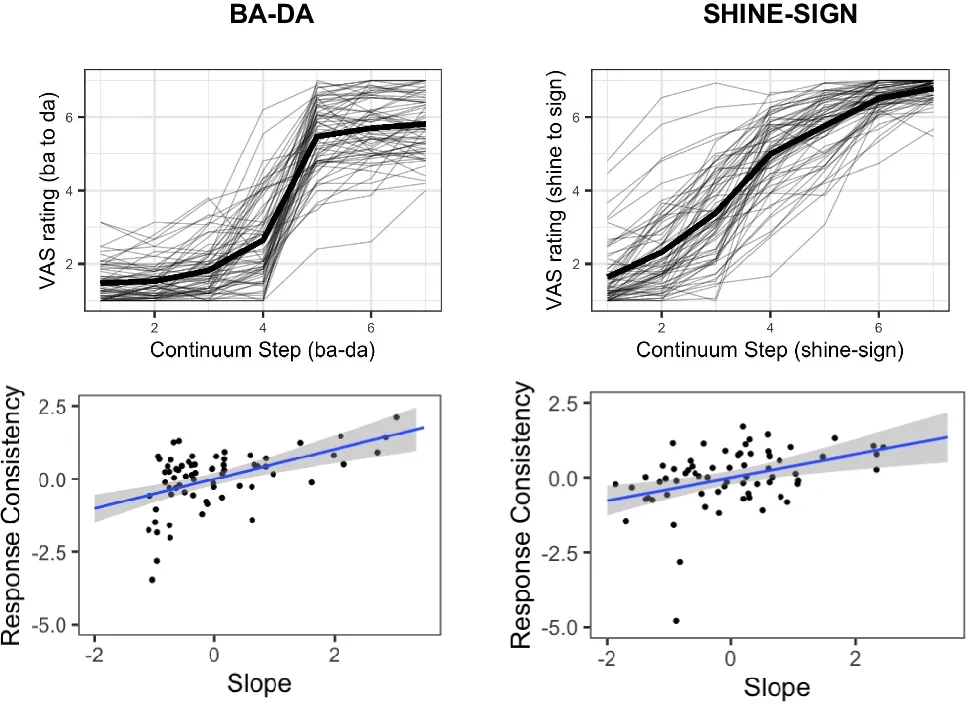
Visual analogue scale (VAS) data for both continua. In the top row, we show the response data. The continuum step is along the x-axis, and the y-axis shows the actual rating the participants made. The thicker black line represents the aggregate group data, and each thinner line represents one subject’s response data. In the bottom row, we show the relationship between our measures of interest extracted from each subject’s responses. Along the x-axis we show the scaled slope values extracted from our mixed effects model. Along the y-axis, we show the scaled response consistency calculated as the average standard deviation across each continuum step

**Fig. 4:**
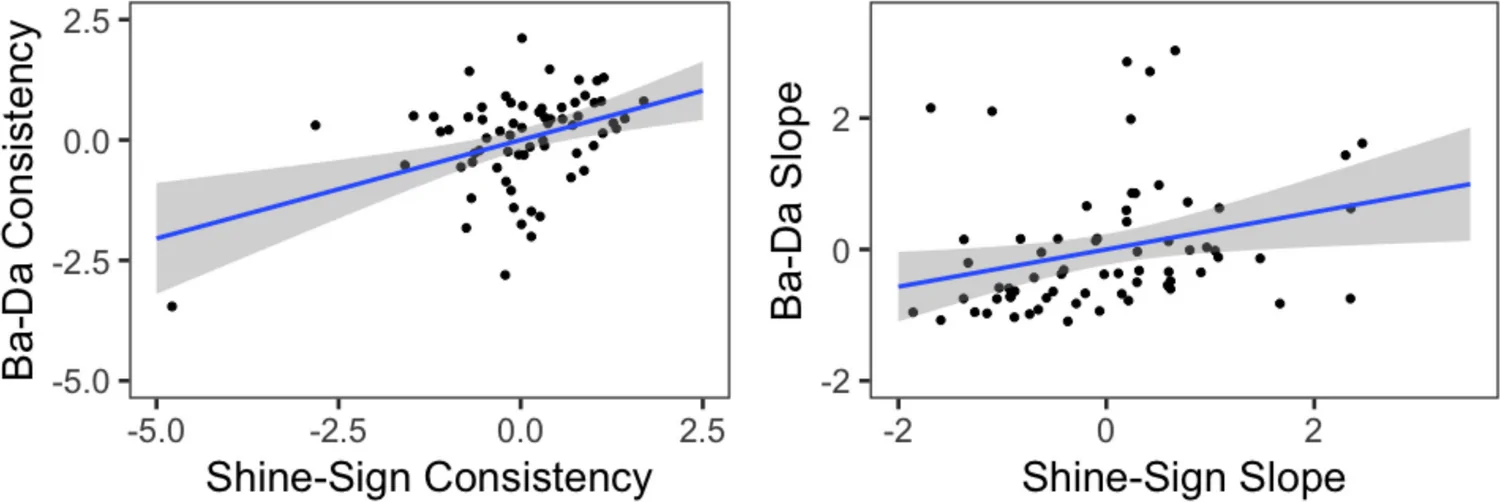
Relationship across visual analogue scale (VAS) continua for our two measures of interest. In both plots, the x-axis shows the scaled values derived from the shine-sign continuum, and the y-axis shows the scaled values derived from the ba-da continuum. The left plot shows the relationship between consistency values across continua, and the right plot shows the relationship between slope values across continua

Participants varied in both their response slope and response consistency (Fig. 3). We found a moderate relationship between slope and response consistency for both continua (ba-da: r = -.507, *t*(65) = −4.740, *p* <.001; shine-sign: r = -.385, *t*(65) = −3.368, *p* =.001). Individuals with higher slopes (i.e., more categorical in their response) tended to have higher consistency. Additionally (Fig. 4), we found moderate relationships within measures across continua (slopes: r = 0.284, *t*(65) = 2.388, *p* =.020; consistencies: r = 0.409, *t*(65) = 3.613, *p* <.001).

### Semantic priming task

**Fig. 5:**
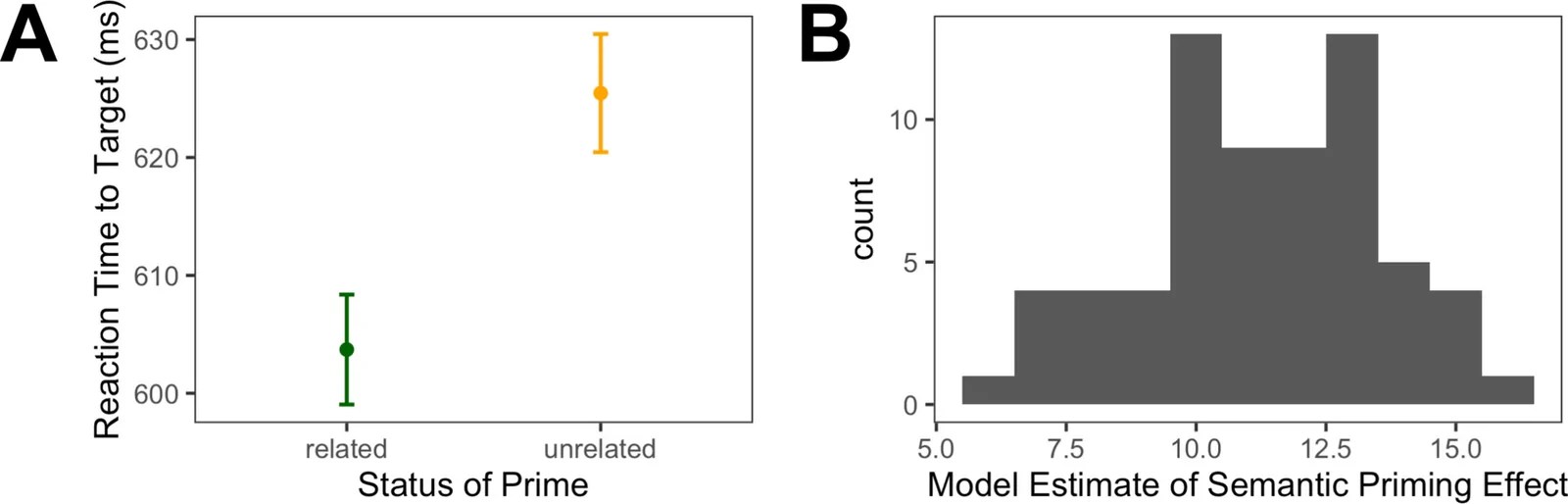
A. Results from the semantic-priming task. The y-axis shows the reaction time to the target item in ms. The x-axis shows the status of the prime word, or whether the word was related or unrelated to the target word. Error bars show 95% confidence intervals. Participants were faster to respond when the prime was related to the target than when the prime was unrelated to the target. **B.** Model estimates of semantic priming effects. This histogram shows the distribution of random slopes for the effect of the status of the prime as extracted from our mixed-effects model

Group-level data showed prime sensitivity (Fig. 5A), with faster responses after related than unrelated primes. The mixed-effects model revealed significantly faster responses with higher frequency (χ2 = 40.00, *p* <.001), trial number (χ2 = 35.68, *p* <.001), and prime status (χ2 = 31.73, *p* <.001). Individual participants were differentially sensitive to the status of the prime (Fig. 5B).

### Across-task relationships

We examined correlations to assess whether our independent measures of semantic and acoustic–phonetic sensitivity related to cue weighting in the sentence task. We predicted that individual differences in semantic priming would predict the strength of semantic cue use in the cue-weighting task, and that individual differences in slope and response consistency in the VAS task would predict individual differences in the use of acoustic–phonetic cues (using vowel length as a predictor, since the use of vowel length and rate were highly correlated, see Fig. 2c).^1^

#### Semantic relationships

**Fig. 6:**
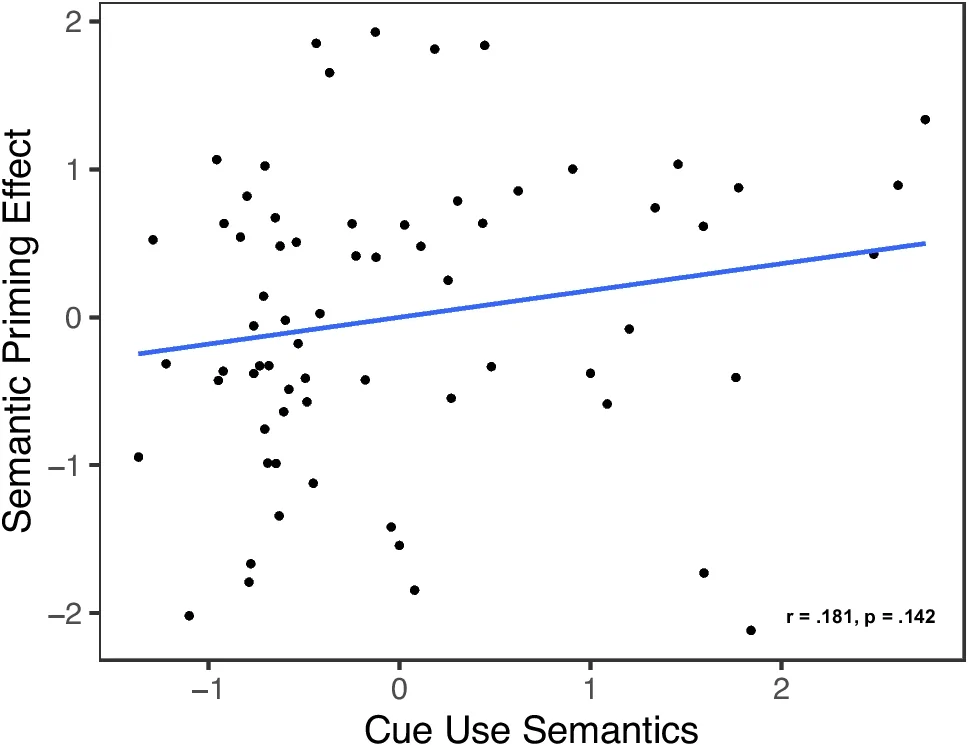
Relationship between our two measures reflecting semantic reliance. The x-axis shows the scaled value of each participant’s slope for reliance on semantic information in the cue-weighting task, and the y-axis shows the scaled value of each participant’s slope for the degree of semantic priming observed in the semantic-priming task

We observed a small positive trend between reliance on semantic information in our cue-weighting task and individual semantic priming (Fig. 6), suggesting that individuals who relied more on semantic information in one task were similarly influenced by it in the other. However, this relationship was not statistically significant (r = 0.181, *t*(65) = 1.485, *p* =.142).

#### Acoustic–phonetic relationships

**Fig. 7:**
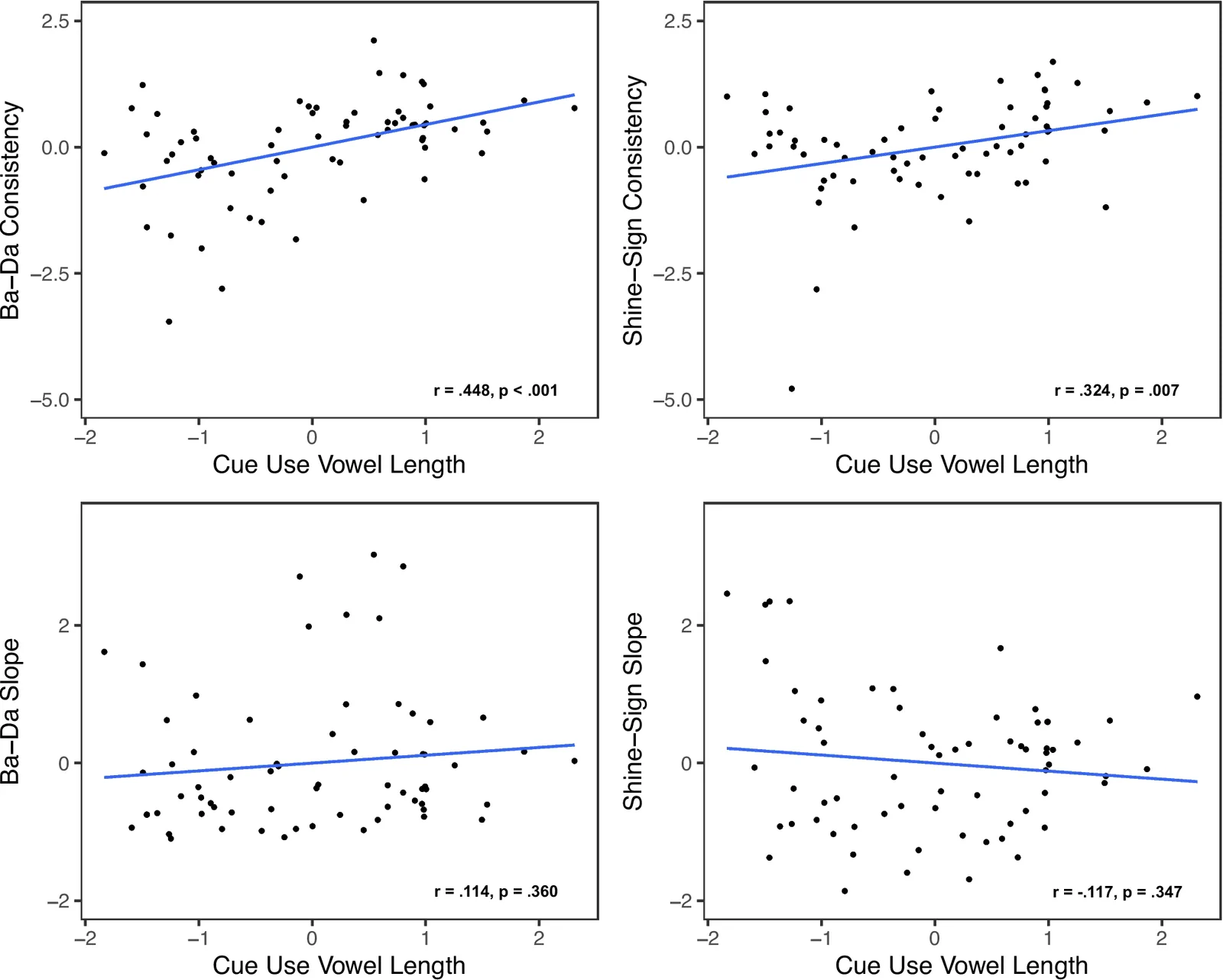
Relationship between our measures reflecting acoustic–phonetic cue reliance across tasks. In the top row, the two graphs display our cue use measure of acoustic–phonetic reliance along the x-axis, and our consistency measures along the y-axis. In the bottom row, the two graphs display our cue use measure of acoustic–phonetic reliance along the x-axis, and our slope measures along the y-axis. All measures are plotted as their scaled values

We observed a positive relationship between reliance on our acoustic–phonetic cue (vowel length) in the cue-weighting task and consistency across both VAS continua (Fig. 7). These relationships are statistically significant (ba-da: r =.448, *t*(65) = 4.040, *p* <.001; shine-sign: r =.324, *t*(65) = 2.761, *p* =.007), indicating that individuals who tend to rely on acoustic–phonetic information in the cue-weighting task tend to be more consistent responders in the VAS task.

Additionally, as shown in Fig. 7, we *did not* observe significant relationships between acoustic–phonetic cue weighting in the cue-weighting task and VAS slopes (ba-da: r =.114, *t*(65) = 0.922, *p* =.360; shine-sign: r = -.117, *t*(65) = −0.948, *p* =.347).

## Discussion

Across three tasks, we assessed the degree of reliance on higher-level cues (semantic information) and lower-level cues (acoustic–phonetic). While recent research suggests individuals reliably differ in reliance on different cues (Crinnion et al., 2024a; Giovannone & Theodore, 2023; Kaufeld et al., 2020), this evidence primarily comes from tasks where cues conflict with each other, potentially “forcing” participants to “choose” to attend to one type of cue. In this study, we tested whether cue-use patterns related to two separate tasks that independently assessed acoustic–phonetic (VAS task) and semantic (semantic-priming task) cue use. This allows us to infer whether individual differences in semantic and acoustic–phonetic sensitivity are chronic individual tendencies that may apply across tasks and contexts.

We first replicated a tradeoff between reliance on acoustic–phonetic and semantic cues (Crinnion et al., 2024a) in our cue-weighting task. Participants who relied more on semantic cues tended to rely less on acoustic–phonetic cues and vice versa. Further, participants’ use of acoustic–phonetic cues (vowel length and speech rate) patterned closely together.

More importantly, we found that the degree of reliance on acoustic–phonetic cues related to a listener’s independently assessed acoustic–phonetic sensitivity (indexed by consistency in VAS ratings of two phonetic continua). Our finding that phonetic response consistency correlates with use of acoustic–phonetic cues even when there are other available cues suggests that highly consistent listeners may more precisely encode acoustic–phonetic detail. Participants with lower reliance on acoustic–phonetic information tended to have lower VAS consistency and exhibited stronger use of top-down information. This converges with findings from other tasks that suggest reliance on higher-level cues may emerge as a compensatory mechanism for underlying difficulties accessing acoustic–phonetic detail (Giovannone & Theodore, 2021b; Koeritzer et al., 2018; Lam et al., 2017; Sheldon et al., 2008).

It is also notable that VAS response consistency and not slope (which relates to how categorically a listener responds) predicts phonetic cue reliance (although see Kapnoula et al., 2021, among others for associations between phonetic category slope and differences in lexical access; also note that we observed a weak positive correlation between slope and consistency, so more consistent responders *tend* also to be more categorical; see Apfelbaum et al., 2022, for discussion of relations between these measures). We hypothesize that response consistency is an index of encoding fidelity, with positive impact on perception and learning. More consistent responders tend to be better at perceiving speech in noise (Myers et al., 2024) and learning non-native speech contrasts (Fuhrmeister et al., 2023). In this study we add to this evidence to show that consistent listeners tend to rely more on acoustic–phonetic cues when listening to sentences with multiple and sometimes conflicting cues. The ability to perceive and attend to the most relevant cues in the input is likely to be helpful in many situations, but especially in second-language acquisition (e.g., Yazawa et al., 2020). The failure to find a relationship between the slope of the VAS function and the cue-weighting task is consistent with prior reports showing that response consistency, but not slope, is associated with a variety of language skills, and especially tasks that require attention to acoustic signal (Fuhrmeister et al., 2023; Kim, et al., 2025; Kutlu et al., 2024; Myers et al., 2024). In essence, it may matter less precisely *how* listeners perceive the structure of a phonetic category, but rather that they do so in a reliable and consistent way.

We opted not to test sensitivity to the precise acoustic–phonetic contrast used in the cue-weighting task, which was a word-final stop voicing contrast.^2^ This decision was made because of concerns about carry-over effects between tasks, and out of a desire to assess general, trait-level differences in phonetic perception that transcended a specific contrast. With this dataset we are unable to evaluate the possibility that differences in sensitivity to a particular acoustic–phonetic dimension (stop voicing) might affect sensitivity to that same contrast in the sentence-listening task. The fact that VAS consistency from two rather different phonetic contrasts continues to predict acoustic–phonetic reliance on a third phonetic dimension in the cue-weighting task points to an individual difference in acoustic–phonetic perception that transcends sensitivity to a particular acoustic dimension.

Some caveats are in order. The current study relied on an online sample, and as such we do not have information about hearing status beyond self-report, and the reliance on a headphone check. As such, differences in the audibility of the acoustic information in the VAS task might have influenced response patterns, potentially leading to less consistent responses in this task as well as greater reliance on semantic information in the cue-weighting task. This relationship awaits confirmation in a well-controlled sample with better audiological assessment.

In contrast to the strong connection between response consistency and acoustic–phonetic cue weighting, we did not observe a significant relationship between semantic priming and reliance on semantic information in the cue-weighting task. There are two potential reasons for this lack of relationship. First, internal reliability of the semantic-priming task is potentially lower than that for VAS, which constrains the strength of any correlation with another task (Hedge et al., 2018). Second, giving greater weight to semantic information in the cue-weighting task may also follow from acoustic–phonetic encoding fidelity rather than an independent tendency or bias towards semantic reliance. That is, individuals who rely more on semantic information may be doing so to compensate for lower encoding ability rather than because of a relative strength in semantic processing. This reasoning would be consistent with findings that individuals with weaker language abilities rely more on lexical as opposed to phonetic information (Giovannone & Theodore, 2021a), among other work suggesting that reliance on higher-level context may be compensatory (e.g., Lam et al., 2017). However, it is possible that the causality could be reversed: individuals might less precisely encode phonetic details *because* they rely more on semantic context, for example. Disentangling these possibilities is a challenge for future research.

In summary then, cue reliance when listening to sentences may be primarily determined by how well listeners access or reliably represent the low-level acoustic–phonetic signal. An open question is how individual differences in bottom-up and top-down cue reliance relate to other perceptual capacities (e.g., auditory acuity) and non-linguistic cognitive capacities. Future work can examine whether individual differences manifest similarly in populations expected to differentially rely on top-down versus bottom-up information. For instance, some literature suggests that older adults rely more on higher-level context (Laver & Burke, 1993; Pichora-Fuller et al., 1995; Sommers & Danielson, 1999), though the evidence is mixed (e.g., Bieber et al., 2022; Sheldon et al., 2008).

Methodologically, this study adds to the growing literature establishing construct validity in psycholinguistic tasks (Heffner & Myers, 2021). For example, Giovannone and Theodore (2023) found weak evidence of relationships across different tasks thought to measure similar reliance on higher- versus lower-level cues. Future work should therefore more carefully examine how performance on tasks like our cue-weighting task relates to a wider range of other measures (such as cognitive and hearing factors).

Our findings again highlight that these individual differences in reliance on different types of information are meaningful and should be considered across a wide range of speech perception and language tasks. If individuals who rely more on semantic information when listening to sentences in our cue-weighting task do so because they have less stability in accessing the acoustic–phonetic signal, then theories of how different types of information influence speech perception need to take into account underlying individual differences in the ability to access the type of information in question.

## Data Availability

Data are available at: https://osf.io/dzb4a/?view_only=2eb90249a8354150b8934eb41afa0f09. Stimulus materials are available upon request to the authors.

## Footnotes

1 Although opinions differ about best practices for outlier removal, we wanted to ensure that no results were driven by extreme values. To this end, we re-ran analyses with responses greater than 3 *SD* from the mean removed (see Berger & Kiefer, 2021, for recommendation of this approach). Six data points were identified as potential outliers, and re-running analyses with pairwise removal of these values did not change the significance of any statistical test.

2 Notably, fricative continua tend to be more continuously perceived than stop continua (e.g., Fry et al., 1962; Healy & Repp, 1982; Massaro & Cohen, 1983). Longer-duration sounds (e.g., vowels and fricatives) may allow listeners more time to encode acoustic differences between tokens, leading to greater within-category sensitivity (Crowder, 1973; Pisoni, 1971). Alternatively, differences in the perceptibility of particular contrasts stem from between-talker variability in production (Newman et al., 2001). More generally, if a contrast exhibits substantial between-talker variability, listeners may develop more gradient sensitivity to it (McMurray & Jongman, 2011).

## Code availability

Analysis code is available at: https://osf.io/dzb4a/?view_only=2eb90249a8354150b8934eb41afa0f09.

## Declarations

### Open practices

This study was not pre-registered. Data and code are available at https://osf.io/dzb4a/?view_only=2eb90249a8354150b8934eb41afa0f09. Experimental materials (stimuli) are available upon request.
